# Identification of mitochondrial carrier homolog 2 as an important therapeutic target of castration-resistant prostate cancer

**DOI:** 10.1038/s41419-025-07406-5

**Published:** 2025-02-05

**Authors:** Yankui Liu, Anjie Chen, Yufan Wu, Jiang Ni, Rong Wang, Yong Mao, Ning Sun, Yuanyuan Mi

**Affiliations:** 1 Department of Pathology, Affiliated Hospital of Jiangnan University, Wuxi, China; 2 Wuxi School of Medicine, Jiangnan University, Wuxi, China; 3 Department of Urology, Affiliated Hospital of Jiangnan University, Wuxi, China; 4 Department of Urology, Kunshan Hospital of Traditional Chinese Medicine, Kunshan, China; 5 Department of Pharmacy, Affiliated Hospital of Jiangnan University, Wuxi, China; 6 Department of Oncology, Affiliated Hospital of Jiangnan University, Wuxi, China

**Keywords:** Prostate cancer, Oncogenes

## Abstract

We here investigate the expression of the mitochondrial carrier homolog 2 (MTCH2) and its potential function in castration-resistant prostate cancer (CRPC). Bioinformatic analyses reveal that *MTCH2* overexpression is associated with critical clinical parameters of prostate cancer. Single-cell sequencing data indicate elevated *MTCH2* expression in the prostate cancer epithelium. MTCH2 is also upregulated in locally treated CRPC tissue and various primary human CRPC cells. Using genetic silencing via shRNA and knockout (KO) through the CRISPR-sgRNA approach, we showed that the depletion of MTCH2 impaired mitochondrial function, resulting in a reduced oxygen consumption rate, diminished complex I activity, and decreased ATP levels, mitochondrial depolarization, and increased reactive oxygen species production in primary CRPC cells. The silencing or KO of MTCH2 significantly inhibited cell viability, proliferation, and migration, together with a marked increase in apoptosis in the primary CRPC cells. In contrast, ectopic expression of MTCH2 provided CRPC cells with pro-tumorigenic properties, enhancing ATP production and promoting cell proliferation and migration. MTCH2 silencing also markedly inhibited the growth of subcutaneous xenografts of the primary CRPC cells in nude mice. The MTCH2-silenced xenografts exhibited increased apoptosis, elevated lipid peroxidation, and decreased ATP levels. These results provide new insights into the role of MTCH2 in supporting mitochondrial function and CRPC progression.

## Introduction

Prostate cancer is among the most prevalent malignancies affecting the male demographic on a global scale [1, 2]. In developed nations, the incidence of prostate cancer surpasses that of all other cancers in men, making it the most frequently diagnosed malignancy in this group [1, 2]. In the United States, the mortality rate associated with prostate cancer is relatively high, ranking second only to lung cancer among male oncological deaths [1, 2]. Furthermore, in emerging economies such as China, there has been a marked increase in the incidence of prostate cancer in recent years [3, 4]. A significant proportion of these cases are diagnosed at advanced stages [5], with many patients presenting in the middle to late stages of the disease at the time of diagnosis [3, 4]. This trend underscores the urgent need for enhanced early detection and intervention strategies in both developed and developing regions [3, 4].

Current treatments, such as prostatectomy, androgen-deprivation therapy, and radiotherapy, can temporarily manage and improve the condition of patients with advanced prostate cancer. However, these interventions are effective only for limited durations [3, 4]. After a median remission period of 18 to 24 months, patients with prostate cancer may progress to metastatic castration-resistant prostate cancer (CRPC) [3, 4]. The median survival for CRPC patients is ~12 months [3, 4, 6, 7]. Docetaxel is the first-line chemotherapeutic agent for CRPC, but many patients eventually develop resistance [3, 4]. The US Food and Drug Administration has approved new endocrine therapies, including enzalutamide and abiraterone, for use in CRPC patients either prior to or following docetaxel chemotherapy failure [8–10]. Despite these advances, the overall prognosis for CRPC remains suboptimal [8–10].

Mitochondrial function is integral to the pathophysiology, progression, and therapeutic resistance of CRPC. CRPC cells exhibit significant metabolic adaptations, such as augmented oxidative phosphorylation (OXPHOS) and enhanced fatty acid oxidation, to fulfill their increased energy demands [11–15]. This metabolic flexibility underpins cell proliferation in the context of androgen-deprivation therapy [11–15]. Furthermore, mitochondrial production of reactive oxygen species (ROS) fosters genetic instability, thereby driving cancer progression [11–15]. Mitochondria also regulate apoptosis, with CRPC cells often upregulating anti-apoptotic proteins to circumvent programmed cell death [11–15]. Consequently, targeting mitochondrial bioenergetics and dynamics presents a compelling and novel therapeutic approach to overcoming resistance and enhancing treatment efficacy in CRPC [11–15].

Mitochondrial carrier homolog 2 (MTCH2), a member of the solute carrier 25 family, diverges from its typical role of substrate exchange across the inner mitochondrial membrane (IMM) by primarily localizing to the outer mitochondrial membrane (OMM) [16, 17]. It functions as an insertase, inserting cytoplasmic α-helical proteins into the OMM, and as a scramblase, facilitating the translocation of phospholipids between mitochondrial membrane leaflets, thereby contributing to mitochondrial dynamics and function [16, 17]. MTCH2 interacts with pro-apoptotic proteins such as truncated BID to facilitate translocation to the mitochondria, thereby regulating mitochondrial outer membrane permeabilization and apoptosis [18]. Recent studies have uncovered a critical role of MTCH2 in both physiological and pathological contexts, impacting metabolic diseases, neurodegenerative disorders, cancers, embryonic development, and reproduction [17].

Studies have suggested that MTCH2 plays an oncogenic role in several cancers. Yuan et al. have shown that MTCH2 is overexpressed in glioma, correlating with poor prognosis and overall survival [19]. MTCH2 silencing disrupted mitochondrial functions, induced oxidative damage, hindered cell migration/invasion, inhibited pro-survival AKT signaling, and increased temozolomide sensitivity in glioma cells [19]. Jiang et al. reported that MTCH2 expression is upregulated in breast cancer and enhances cellular proliferation and cell cycle progression through the PI3K-Akt pathway [20]. Li et al. showed that apolipoprotein C1 facilitated the progression of osteosarcoma by interacting with MTCH2 [21]. The current study focuses on investigating the expression profiles and functional significance of MTCH2 in CRPC, with the goal of elucidating its potential as both a biomarker and a therapeutic target.

## Methods

### Reagents

Antibodies were obtained from Abcam Co. (Shanghai, China). The viral constructs and the mRNA primers were provided by Genechem (Shanghai, China). The cell counting kit-8 (CCK-8) was purchased from Sigma (St. Louis, MO). Promega (Shanghai, China) supplied the GSH/GSSG ratio kit, the mitochondrial Complex I activity assay kit, and the ATP assay kit. All fluorescent dyes were acquired from Thermo Fisher Invitrogen (Suzhou, China).

### Primary human cell isolation and cultivation

As reported earlier [22], tumor and adjacent epithelial tissue from CRPC patients were excised and minced before enzymatic digestion with collagenase I and dispase II. The resultant cell suspension was cultured in the complete medium, followed by filtration. Non-adherent cells, including the endothelial cells, fibroblasts, and immune cells, were discarded. The isolated primary cancer cells and prostate epithelial cells were cultured as described [23]. Specifically, primary CRPC cells (“pPC-1”, “pPC-2”, “pPC-3”, and “pPC-4”) from four patients and primary prostate epithelial cells (“pEpi1” and “pEpi2”) from two patients were established in an earlier study [22]. All procedures involving human cells received approval from the Ethics Committee of the Affiliated Hospital of Jiangnan University, in accordance with the Declaration of Helsinki.

### Human tissue

As reported earlier [22], a group of fifteen (15) male CRPC patients, aged 59 to 82, from the Affiliated Hospital of Jiangnan University were included in this study with written-informed consent. Fresh CRPC tissue and paired cancer-surrounding normal prostate tissue were collected and preserved in liquid nitrogen for subsequent analysis. These protocols were approved by the Ethics Committee of the Affiliated Hospital of Jiangnan University, adhering to the Declaration of Helsinki.

### Western blotting

Following the established protocols [22, 24], protein lysates (30–40 µg) from cells and tissue were separated on SDS-PAGE gels (10–12.5%) and transferred to polyvinylidene fluoride (PVDF) membranes. Membranes were blocked and incubated overnight at 4 °C with specific primary antibodies, followed by 50–60 min at room temperature with secondary antibodies. Protein bands were visualized under enhanced chemiluminescence (ECL) and quantified via ImageJ software. The mitochondrial lysates were isolated by centrifuging the protein lysate supernatant at 11,000× *g* for 12 min to pellet the mitochondria, followed by re-suspending the pellet in the Mitochondria Storage Buffer (Sigma). The uncropped blotting images are listed in Fig. S1. GAPDH or Tubulin protein expression was assessed as a loading control.

### Quantitative real-time PCR (qPCR)

According to previous methods [22, 24], RNA was extracted from cells and tissue using TRIzol, reverse transcribed into cDNA with a Takara PCR amplification kit (Beijing, China), and analyzed by qPCR via SYBR Green PCR Master Mixes (Beijing, China) under the ABI-7900 system. *GAPDH* was used as the reference gene, and relative gene expression was calculated using the 2^−ΔΔCt^ method. The primers were provided by Genechem.

### MTCH2 silencing

Three distinct shRNAs targeting MTCH2, designated as “shMTCH2-S1”, “shMTCH2-S2”, and “shMTCH2-S3” (targeting non-overlapping sequences), along with a scramble control shRNA (“c-sh”, as a control), were cloned into the GV369 vector from Genechem. The lentiviruses were generated by co-transfecting these constructs with viral packaging plasmids into HEK-293 cells. The resulting viral particles were subsequently used to infect primary cultured prostate cancer cells or epithelial cells. Following a 48 h incubation, the cells were cultured in a fresh complete medium supplemented with puromycin. Stable cells with silenced MTCH2 expression were established after five passages, and MTCH2 knockdown was verified by qPCR and Western blotting assays.

### MTCH2 knockout (KO)

CRPC cells at 50–60% confluence were cultured in a complete medium with polybrene and infected with a Cas9-expressing lentivirus (provided by Dr. Cao [25, 26]) to establish stable cells. These cells were subsequently transduced with a CRISPR/Cas9-MTCH2 KO lentiviral construct containing the small guide RNA (sgRNA) targeting MTCH2, also provided by Genechem). Post puromycin selection, cells were plated at a single-cell density into 96-well plates. Successful MTCH2 KO was confirmed through sequencing of the target region and Western blot analysis. Two stable MTCH2 KO single-cell clones, koMTCH2-cln1 and koMTCH2-cln2, were established, both demonstrating complete depletion of MTCH2 protein. Control cells were transduced with a CRISPR/Cas9-empty vector lentivirus (“koC”), also provided by Dr. Cao [25, 26].

### MTCH2 overexpression

The MTCH2-expressing lentiviral GV369 construct and the vector control (“Vec”, as the control) were obtained from Genechem. Lentiviruses were produced by co-transfecting these constructs with viral packaging plasmids into HEK-293 cells. The resultant viral particles were then used to infect primary cultured prostate cancer cells. After 48 h incubation, the cells were transferred to a fresh complete medium with puromycin. The stable cells overexpressing MTCH2 were established after 4–5 passages, and the overexpression was confirmed by the qPCR and Western blotting assays.

### Viability and colony formation assays

As reported [22, 24], cells with the designated genetic treatments, at 3000 cells per well, were seeded in 96-well plates and incubated for specified time periods. CCK-8 reagent (15 µL/well) was added for 2 h, and absorbance at 450 nm was measured using a microplate reader. For the colony formation experiments, cells were initially seeded at a density of 20,000 cells per well in a 10-cm culture dish. The cells were maintained in the complete medium supplemented with 10% serum, with the medium being renewed every 2 days to ensure optimal growth conditions. After a period of 10 days, the cell colonies were fixed, stained for visualization, and manually counted to assess the number of colonies formed.

### Nuclear TUNEL/EdU Staining

As reported earlier [22, 24], cells with the designated genetic treatments were placed on coverslips in 24-well plates and incubated for the designated time durations. Cells were fixed with 4% formaldehyde for 9 min, then permeabilized with 0.15% Triton X-100 in PBS for 6 min. Nuclei were stained using TUNEL, EdU, or DAPI dyes, and fluorescent images were captured with a fluorescence microscope.

### “Transwell” assays

As reported earlier [22, 24], “Transwell” chambers (Corning, NY) were utilized, with a 300 μL of cell suspension containing 10,000 cells added to each chamber. The lower chamber contained 600 μL of the medium with 10% FBS. After 24 h of incubation, non-migrated cells were removed, and migrated cells were stained with 0.25% crystal violet for 8 min, rinsed with PBS, and photographed. For the in vitro cell invasion assays, “Transwell” inserts were coated with Matrigel (Sigma, at 80 µg/cm²).

### Caspase-3/-9 activity assay

The detailed procedures were described earlier [22, 24]. In brief, total cell or tissue lysates were analyzed using Caspase fluorescent assay kits (BD Bioscience, Suzhou, China). Caspase-3/9 activity was measured fluorometrically with the Ac-DEVD-AMC substrate or the Ac-LEHD-AMC substrate.

### Flow cytometry

Following established protocols [22, 24], cancer cells with designated genetic treatments were cultivated for the designated time, and were centrifuged, resuspended, and stained with Annexin V-APC (10 µL) and/or propidium iodide (PI, 10 µL) (Sigma). Apoptosis was assessed using a CytoFLEX flow cytometer (Beckman, Shanghai, China).

### Histone-DNA ELISA assay

Cells with the designated genetic treatments, at 3000 cells per well, were seeded in 96-well plates and incubated for specified time periods. The histone–DNA ELISA assay quantitatively measures histone-associated DNA fragments, which are the specific markers of cell apoptosis. The antibody in the kit captured histone-DNA complexes from samples, which were then detected by an enzyme-linked secondary antibody producing a colorimetric signal at 450 nm.

### Fluorescence dye assays measuring mitochondrial functions

After incubation for the designated time periods, CRPC cells were fixed with methanol, permeabilized with Triton X-100, blocked with BSA, and washed with PBS. Fluorescence dyes, including CellROX, JC-1, and Mito-SOX (all provided by Thermo Fisher, Suzhou, China), were then applied. Fluorescent images were captured using a Zeiss microscope, with relative fluorescence intensity quantified through the ImageJ software.

### Mitochondrial complex I activity and ATP assays

The activity of mitochondrial complex I in cellular/tissue lysates was evaluated using a Sigma kit, which measures the NADH to NAD+ conversion via spectrophotometry. The reduction in absorbance at 350 nm indicated complex I activity. Cellular and tissue ATP concentrations were determined with a Sigma colorimetric kit, following the standard protocols. For each sample, 30 μL of lysates (including 30 μg of total proteins) were used for analysis.

### Oxygen consumption rate (OCR)

OCR was assessed using the XF24 Extracellular Flux Analyzer (Agilent SeaHorse Bioscience) as per established methods [27]. Cells were sequentially exposed to 1 μM oligomycin, 0.5 μM FCCP (carbonyl cyanide-*p*-trifluoromethoxyphenylhydrazone), and a combination of 0.5 μM antimycin A plus Rotenone to evaluate basal, ATP-linked, maximal, and non-mitochondrial OCR [27]. Measurements were always normalized to intracellular protein concentrations.

### Thiobarbituric acid reactive substance (TBAR) assay of lipid peroxidation levels

The tissue/cellular lysates, each containing 20 μg proteins per sample, were analyzed using a commercial TBAR kit from Cayman Chemical (Ann Arbor, Michigan). This kit specifically quantifies lipid peroxidation and malondialdehyde (MDA) content through a colorimetric method. The TBAR signal intensity was assessed at a wavelength of 555 nm, using 590 nm as the reference.

### GSH/GSSG ratio detection

To measure the ratio of reduced glutathione (GSH) to oxidized glutathione (GSSG), we used a GSH/GSSG ratio kit from Thermo Fisher Scientific (Suzhou, China). Briefly, cell or tissue lysates were mixed with 5,5’-Dithio-bis(2-nitrobenzoic acid) (DTNB), glutathione reductase, and an NADPH mix. Afterward, the lysates were mixed with the reaction solution, and absorbance at 425 nm was measured over 5 min using a spectrophotometer. A standard curve with GSH and GSSG standards determined their concentrations in the lysates, and the ratio was normalized to protein concentration for accurate comparison.

### Xenograft studies

The BALB/c nude mice, with an average weight range of 17.7–18.1 g and equally distributed by sex, were purchased from the Shanghai Laboratory Animal Center (SLAC, Shanghai, China). The primary CRPC cells (eight million per tumor) were subcutaneously injected into the flank of these mice. The formation of the subcutaneous xenograft tumors was observed, and data collection commenced on day 10 (“Day 10”). The tumor volumes, the body weights, and the daily tumor growth rate (in mm³ per day) were measured and calculated according to established protocols [22]. For the immunohistochemistry (IHC) assays, the xenograft tumors were initially fixed, embedded in paraffin, and sectioned into the 4-μm slices. These sections underwent deparaffinization, rehydration, and antigen retrieval processes. After blocking, the sections were incubated with a primary antibody, followed by a secondary antibody, and visualization was achieved using diaminobenzidine (DAB). Additionally, sections were stained using the TUNEL assay (Biyuntian, Wuxi, China), followed by washing and counterstaining with DAPI to highlight the nuclei. The sections were then mounted with an anti-fade medium and examined under a fluorescence microscope. All procedures involving animal experiments were approved by the Institutional Animal Care and Use Committee (IACUC) and the Ethics Review Board of the Affiliated Hospital of Jiangnan University.

### Statistical analysis

All the in vitro experiments were conducted in quintuplicate. Data, consistently normally distributed, are presented as mean ± standard deviation (SD). Statistical analyses were performed using SPSS software (SPSS Inc., Chicago, IL). For comparisons between the two groups, an unpaired Student’s *t*-test was employed. One-way ANOVA followed by Scheffe’ and Tukey tests was used for comparisons among three or more groups. The *P* values less than 0.05 were considered statistically significant.

## Results

### *MTCH2* overexpression correlates with key clinical parameters of prostate cancer

**Fig. 1:**
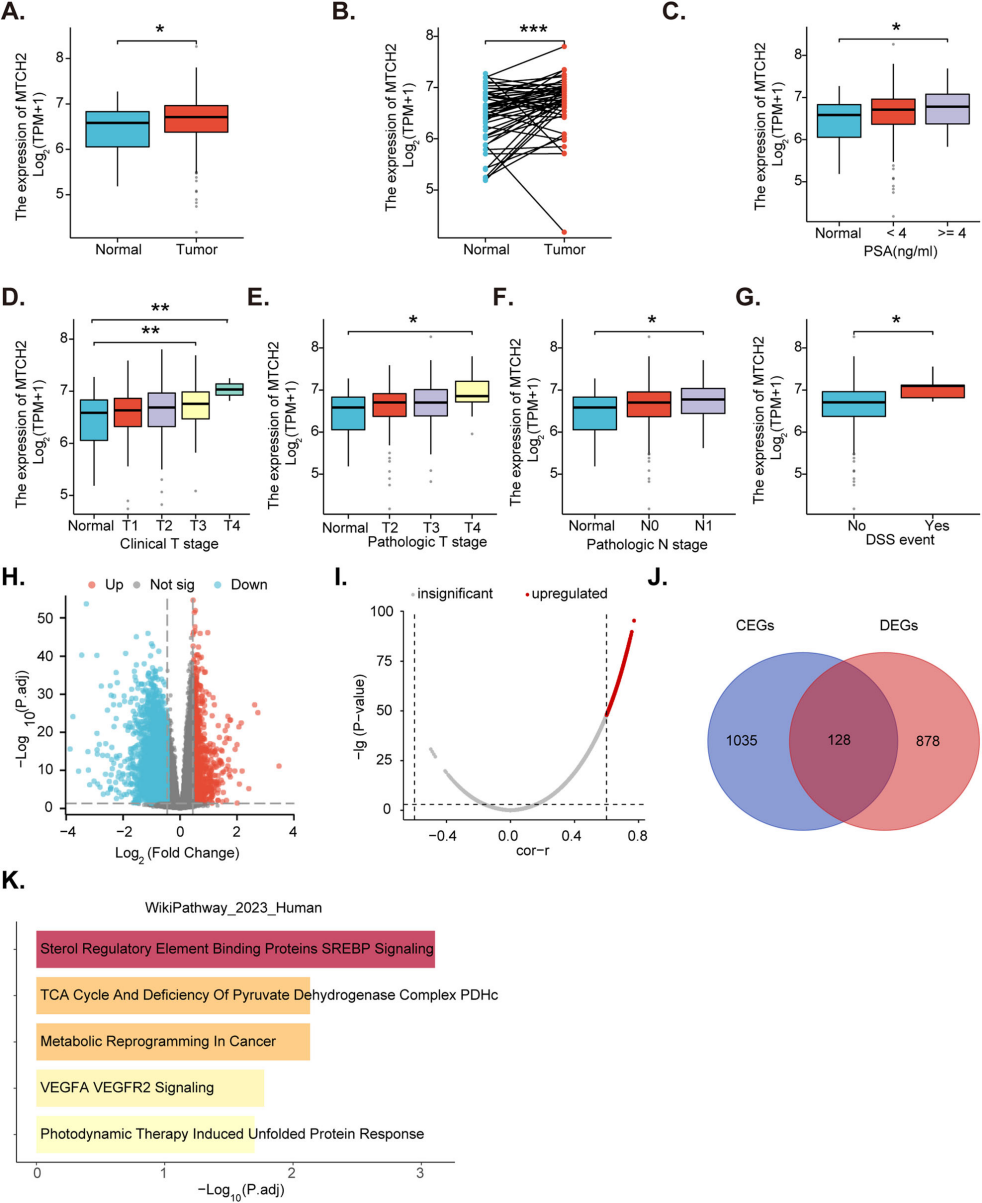
*MTCH2* overexpression correlates with key clinical parameters of prostate cancer. TCGA-PRAD cohort analysis compares the *MTCH2* mRNA transcript levels between prostate cancer tissue (“Tumor”) and normal tissue (“Normal”) from the described patients (**A**–**G**). Correlation analysis identified co-expressed genes (CEGs) with *MTCH2* from TCGA-PRAD samples (**H**). Differential expression analysis was performed on TCGA-PRAD samples divided into high- and low-*MTCH2* expression groups to identify differentially expressed genes (DEGs) (**I**). The intersection of CEGs and DEGs resulted in 128 common genes (**J**). The WikPathway pathway enrichment analysis of these 128 common genes revealed significant associations of MTCH2 with multiple pathways (**K**). “TPM” indicates transcripts per million. *P < 0.05, **P < 0.01, and ***P < 0.001.

We initiated our study by querying The Cancer Genome Atlas Prostate Adenocarcinoma (TCGA-PRAD) database to obtain *MTCH2* expression data. Our analysis demonstrated a higher number of *MTCH2* mRNA transcripts in prostate cancer tissue (“Tumor”) compared to the normal prostate tissue (“Normal”) (Fig. 1A). This finding was further supported by the paired sample analysis, which showed elevated *MTCH2* expression in prostate cancer tissue relative to their corresponding normal tissue (Fig. 1B). Additionally, *MTCH2* expression was higher in prostate cancer tissue with prostate-specific antigen (PSA) levels ≥4 compared to those with PSA levels <4 (Fig. 1C). Subgroup analyses revealed that *MTCH2* overexpression was associated with advanced clinical T-stage (Fig. 1D) and a higher tumor grade (Fig. 1E). Moreover, *MTCH2* expression was elevated in N1-stage prostate cancer tissue compared to N0-stage tissue (Fig. 1F). Importantly, overexpression of *MTCH2* in prostate cancer tissue was correlated with decreased disease-specific survival (DSS) (Fig. 1G).

Using the TCGA-PRAD prostate cancer dataset, we performed a correlation analysis with *MTCH2*, setting thresholds of ***P*** < 0.05 and a correlation coefficient ***R*** > 0.6 to identify co-expressed genes (CEGs) (Fig. 1H). Based on the median expression levels, samples were divided into high- and low-*MTCH2* expression groups. Differential expression analysis between these groups was conducted, applying thresholds of ***P*** < 0.05 and fold change (FC) >1.5 to identify differentially expressed genes (DEGs) (Fig. 1I). The intersection of the CEGs and DEGs yielded 128 common genes (Fig. 1J). Subsequent WikPathway enrichment analysis of these common genes revealed significant associations of *MTCH2* with metabolic reprogramming, tricarboxylic acid cycle (TCA) cycle, and sterol regulatory element binding protein (SREBP) signaling pathways (Fig. 1K) in prostate cancer.

### Single-cell sequencing shows *MTCH2* overexpression in prostate cancer epithelium

**Fig. 2:**
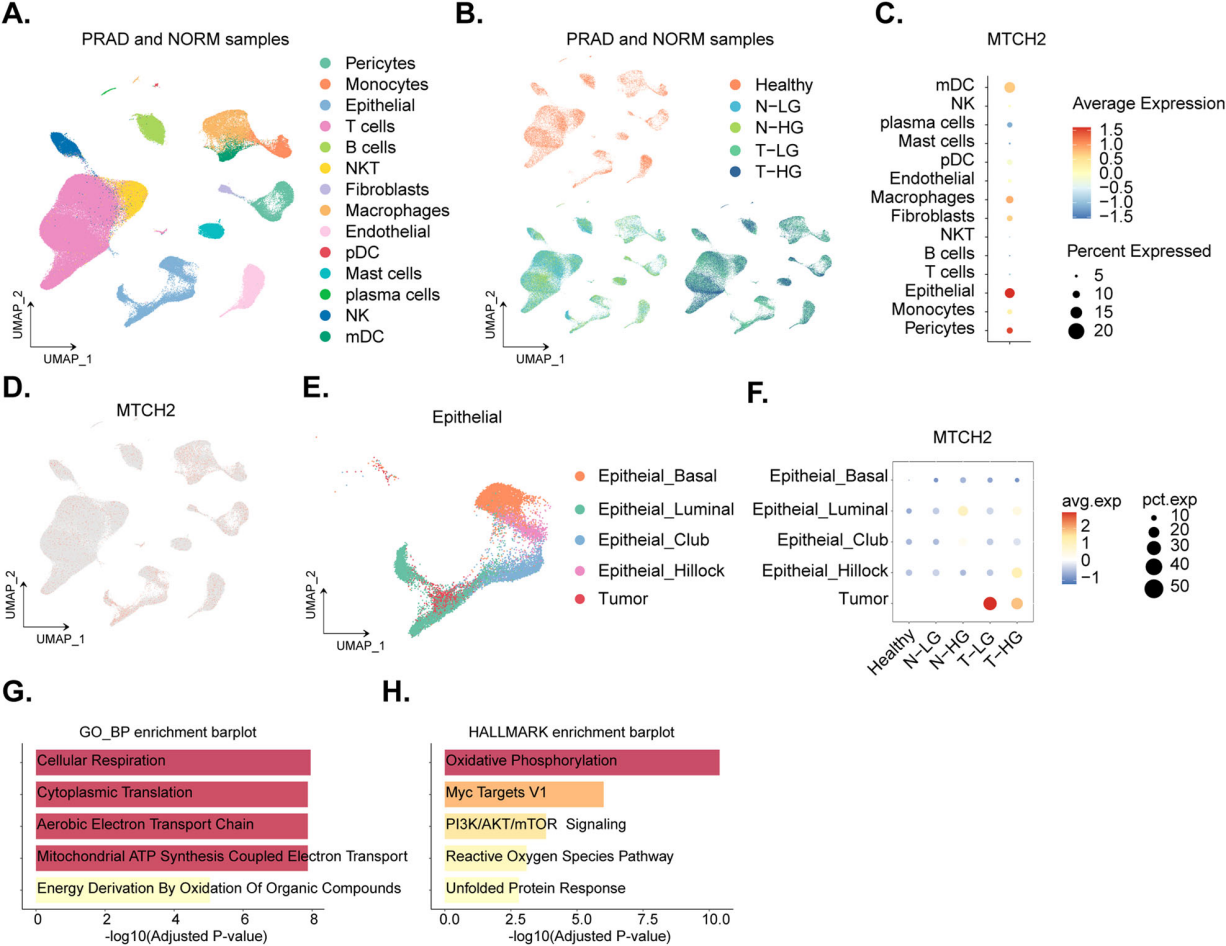
Single-cell sequencing shows *MTCH2* overexpression in the prostate cancer epithelium. Dimensionality reduction visualization showing cell annotations in the GEO single-cell dataset GSE181294 (**A**). A dimensionality reduction visualization depicting described sample groupings (**B**). The dot plot analysis indicates significantly elevated *MTCH2* expression within the epithelial cell population (**C**). The expression density analysis confirmed higher *MTCH2* expression in the epithelial cells of the prostate cancer (“PRAD”) group compared to the normal prostate tissue (“NORM”) group (**D**). Subsequent extraction and sub-clustering of epithelial cells highlighting *MTCH2* distribution in tumor epithelium (**E**). Detailed visualization of *MTCH2* distribution in the tumor epithelium (**F**). Correlation analysis of *MTCH2* expression within the epithelial cells, identifying the top 100 related genes, and Gene Ontology (GO) (**G**) and HALLMARK (**H**) enrichment analyses showing associations of *MTCH2* with biological process and signaling cascades.

A bioinformatics analysis of the Gene Expression Omnibus (GEO) single-cell dataset GSE181294, encompassing prostate cancer (“PARD”/“T”), paired parecancer normal prostate tissue (“NORM”/“N”), and healthy prostate tissue (“Healthy”), was conducted. Cell annotations were provided by the original authors [28]. Dimensionality reduction visualization depicts the cell annotations (Fig. 2A) and sample groupings (Fig. 2B). A dot plot (Fig. 2C) and expression density (Fig. 2D) analyses revealed significantly elevated *MTCH2* expression within the epithelial cell population. Specifically, *MTCH2* expression was significantly higher in the epithelial cells of the prostate cancer (“PRAD”) group compared to the normal prostate tissue (“NORM”) group (Fig. 2C, D). Subsequent extraction and sub-clustering of epithelial cells (Fig. 2E) demonstrated that *MTCH2* was predominantly distributed in tumor epithelium (Fig. 2E, F). Correlation analysis of *MTCH2* expression within epithelial cells identified the top 100 related genes, which were subjected to Gene Ontology (GO) and HALLMARK enrichment analyses. These analyses indicated that *MTCH2* overexpression is closely associated with cellular respiration, the mitochondrial respiratory chain, ATP production, and phosphoinositide 3 kinase (PI3K)-Akt-mammalian target of rapamycin (mTOR) pathway signaling (Fig. 2G, H). These results suggest a role for *MTCH2* in the metabolic and signaling processes of prostate cancer cells.

### Mitochondrial MTCH2 is upregulated in both CRPC tissue and cells

**Fig. 3:**
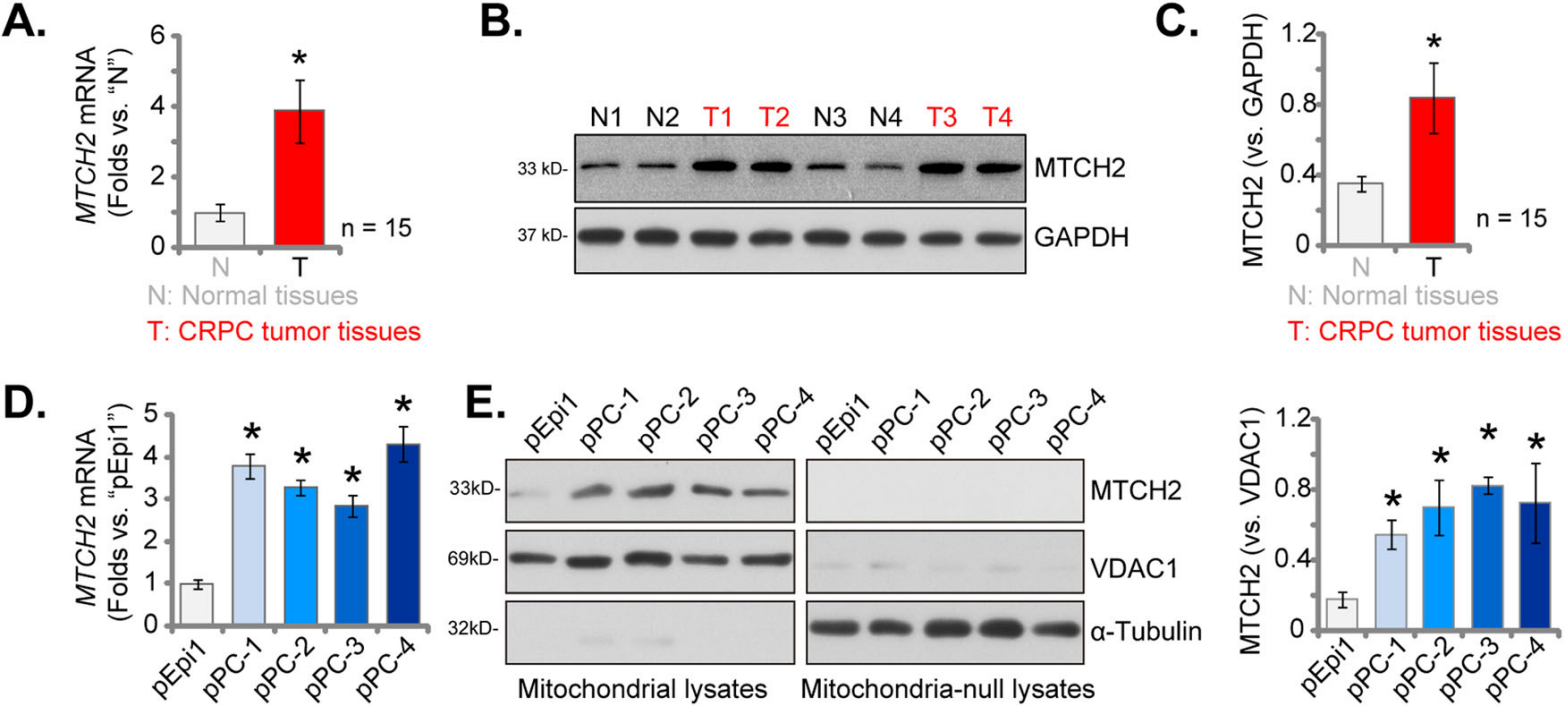
Mitochondrial MTCH2 is upregulated in both CRPC tissue and cells. Expression levels of *MTCH2* mRNA (**A**) and protein (**B**, **C**) in the CRPC tissue (“T”, derived from 15 different CRPC patients) or tumor-surrounding normal prostate tissue (“N”) were shown. *MTCH2* mRNA (**D**) and protein (in both mitochondrial fraction lysates and mitochondria-null lysates, **E**) expression levels in primary prostate cancer cells derived from four CRPC patients (“pPC-1”, “pPC-2”, “pPC-3”, and “pPC-4”) and primary human prostate epithelial cells (“pEpi1”) were shown. The data are expressed as mean ± standard deviation (SD). Statistical significance is marked by *P < 0.05 when compared to “pEpi1” cells or “N” tissue.

We additionally examined MTCH2 expression levels in locally treated CRPC tissue. Analysis of *MTCH2* mRNA via qPCR revealed a significant elevation in human CRPC samples obtained from 15 distinct patients (Fig. 3A). In contrast, levels were comparatively low in the adjacent normal prostate tissue (Fig. 3A). Western blot analysis, as shown in Fig. 3B, corroborated the increased MTCH2 protein expression in CRPC samples from four representative patients (“T1”, “T2”, “T3”, and “T4”). Moreover, a comprehensive quantification of Western blot results across all 15 patient samples confirmed a significant upregulation of MTCH2 protein in CRPC tissue (***P*** < 0.05 compared to normal tissue, Fig. 3C).

Furthermore, we examined the expression levels of MTCH2 in primary prostate cancer cells derived from four CRPC patients, designated as “pPC-1”, “pPC-2”, “pPC-3”, and “pPC-4” [22]. qPCR assays revealed a significant upregulation of *MTCH2* mRNA in all primary CRPC cells (Fig. 3D). In contrast, primary human prostate epithelial cells (referred to as “pEpi1”, as described earlier [22]) exhibited relatively low-*MTCH2* mRNA expression levels (Fig. 3D). Mitochondrial fractions were isolated from both CRPC cells and epithelial cells. The presence of MTCH2 protein was confirmed in the mitochondrial lysates along with the voltage-dependent anion channel 1 (VDAC1), serving as a mitochondrial marker (Fig. 3E). MTCH2 protein levels were elevated in the mitochondrial lysates from primary CRPC cells (Fig. 3E), whereas the protein remained at low levels in epithelial cell lysates (Fig. 3E). Additionally, MTCH2 protein was not detected in lysates devoid of mitochondria, as indicated by the presence of α-Tubulin (Fig. 3E) and the absence of VDAC1 (Fig. 3E). Therefore these results confirm mitochondrial MTCH2 upregulation in both CRPC tissue and cells.

### MTCH2 silencing causes extensive mitochondrial damage in primary human CRPC cells

**Fig. 4:**
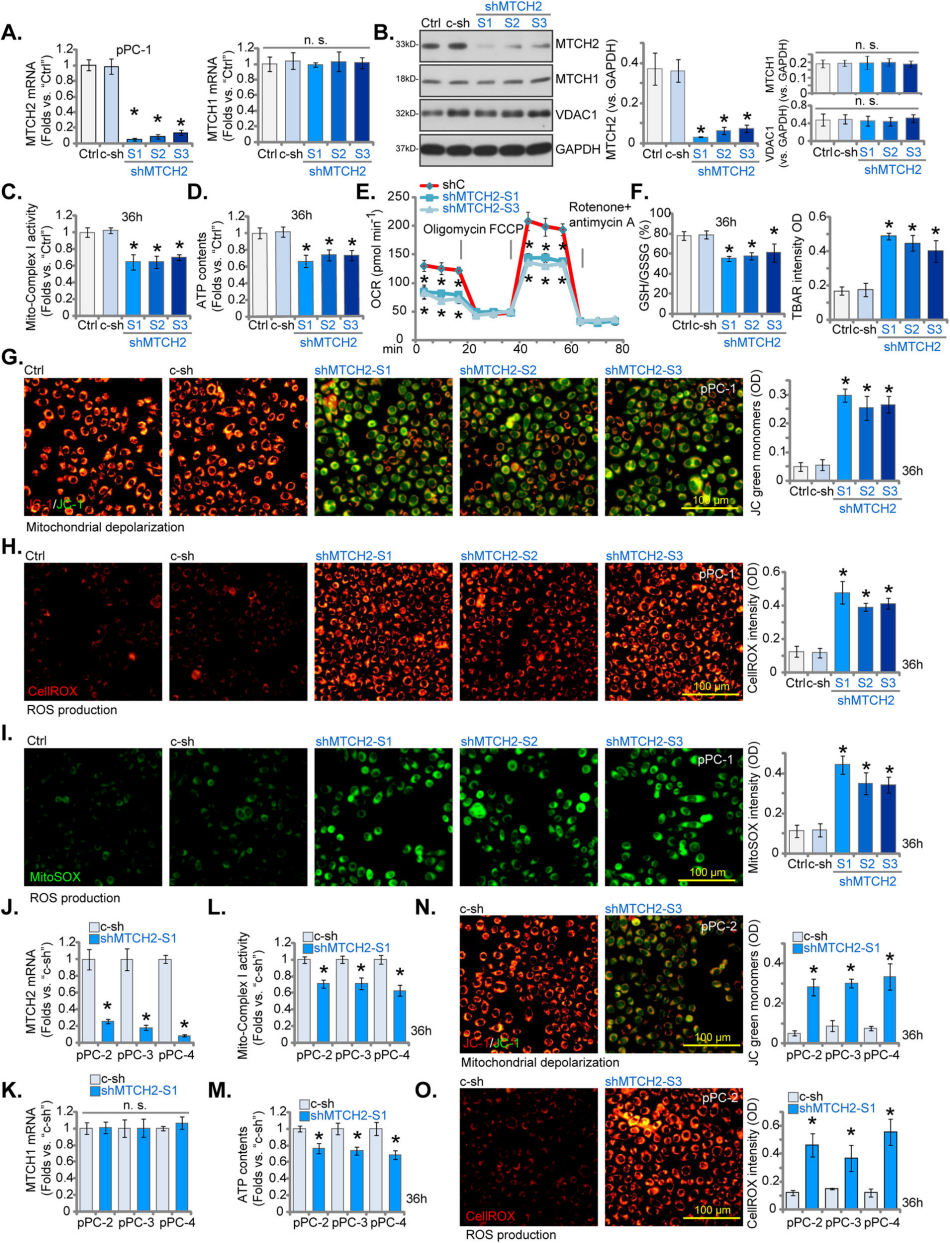
MTCH2 silencing causes extensive mitochondrial damage in primary human CRPC cells. The primary pPC-1 cells were subjected to individual treatments with specific MTCH2 shRNAs (shMTCH2-S1, shMTCH2-S2, or shMTCH2-S3, each representing a unique sequence) or a control scramble non-sense shRNA (c-sh), stable cells were formed after puromycin selection. Expression levels of MTCH1 and MTCH2 were assessed using qPCR (**A**) and Western blotting (**B**) assays. An equal number of cells were cultured for designated time periods, and several key parameters were evaluated, including the mitochondrial complex I activity (**C**), ATP contents (**D**), and oxygen consumption rate (measured using the Seahorse assay, **E**), as well as the GSH/GSSH ratio (**F**, the left panel) and lipid peroxidation. (measured using the TBAR assay, **F**, the right panel). Mitochondrial depolarization was determined by JC-1 green monomer intensity (**G**), and the ROS levels were measured via quantifying CellROX and Mito-SOX fluorescence intensity (**H**, **I**). Additionally, the stable cells derived from other primary CRPC cells (pPC-2, pPC-3, and pPC-4) expressing either c-sh or shMTCH2-S1 were established. The mRNA expression levels of MTCH1 and MTCH2 in these cells were evaluated (**J**, **K**). Cells were cultured for the specific durations, and the mitochondrial complex I activity (**L**), ATP content (**M**), mitochondrial depolarization (tested via JC-1 green monomer intensity, **N**), and ROS production (measured by CellROX intensity, **O**) were tested similarly. The data are expressed as mean ± standard deviation (SD, *n* = 5). “Ctrl” denotes the parental control cells. Statistical significance is marked by *P < 0.05 when compared to “c-sh” cells. “n.s.” denotes non-statistically significant differences (***P*** > 0.05). The experiments depicted in this figure were conducted five times (biological replicates), consistently producing similar results. The scale bar represents 100 μm.

To investigate the functional role of MTCH2 in CRPC cell biology, we utilized an shRNA-mediated knockdown strategy. Lentiviral vectors encoding MTCH2-specific shRNAs were transduced into primary CRPC cells (pPC-1, as previously established [22]), and stable cells were selected using puromycin. We employed three distinct shRNAs targeting MTCH2, labeled as “shMTCH2-S1”, “shMTCH2-S2”, and “shMTCH2-S3”, each with unique sequences to avoid overlap. This approach led to substantial reductions in both *MTCH2* mRNA and protein levels, as evidenced by the quantitative analyses shown in Fig. 4A, B, respectively. In contrast, the expression of *MTCH1* mRNA and protein remained unaffected (Fig. 4A, B). Expression of the mitochondrial protein voltage-dependent anion channel 1 (VDAC1) was also unchanged (Fig. 4B).

In pPC-1 cells, silencing of MTCH2 via targeted shRNAs led to mitochondrial dysfunction. Specifically, we observed a marked reduction in mitochondrial complex I activity in MTCH2-silenced pPC-1 cells (Fig. 4C). This was accompanied by a concomitant decrease in ATP levels, as depicted in Fig. 4D. Results from the Seahorse assay further revealed that the shRNA-mediated suppression of MTCH2 significantly reduced basal and maximal oxygen consumption rates (OCR) in pPC-1 cells (Fig. 4E), suggesting inhibition of mitochondrial respiration. Moreover, the GSH/GSSH ratio was decreased in MTCH2 shRNA-expressing pPC-1 cells (Fig. 4F, the left panel). Silencing of MTCH2 by the applied shRNAs also resulted in a significant increase of TBAR activity in pPC-1 cells, suggesting increased lipid peroxidation (Fig. 4F, the right panel).

Additionally, MTCH2 knockdown led to mitochondrial depolarization, which was demonstrated by a shift in JC-1 fluorescence from red aggregates to green monomers (Fig. 4G). In these MTCH2-silenced pPC-1 cells, we also detected a substantial increase in reactive oxygen species (ROS) production, indicated by heightened CellROX red fluorescence intensity (Fig. 4H) and increased Mito-SOX green fluorescence intensity (Fig. 4I). In contrast, treatment with c-sh did not impair the mitochondrial function in pPC-1 cells (Fig. 4C–I). These findings highlight the extensive mitochondrial damage that arises from MTCH2 silencing in pPC-1 primary CRPC cells.

To evaluate the reproducibility of MTCH2 silencing across various primary CRPC cells, we introduced the lentiviral vector encoding shMTCH2-S1 into other primary human CRPC cells (pPC-2, pPC-3, and pPC-4). The selection of stable cell populations was achieved using puromycin. The experimental results indicated a significant decrease in *MTCH2* mRNA expression levels in the shMTCH2-S1-treated primary CRPC cells (Fig. 4J), while *MTCH1* mRNA levels remained unchanged (Fig. 4K). In these primary CRPC cells, the knockdown of MTCH2 led to a marked reduction in mitochondrial complex I activity (Fig. 4L) as well as in cellular ATP levels (Fig. 4M). The observation of JC-1 green fluorescent monomer accumulation provided additional evidence of mitochondrial depolarization within these cells (Fig. 4N). Furthermore, an increase in CellROX intensity was indicative of enhanced ROS production and oxidative stress in MTCH2-silenced CRPC cells (Fig. 4O). These consistent outcomes across multiple CRPC cells highlight the essential role of MTCH2 in preserving mitochondrial functionality.

### MTCH2 silencing inhibits the proliferative and migratory capacities of primary human CRPC cells

**Fig. 5:**
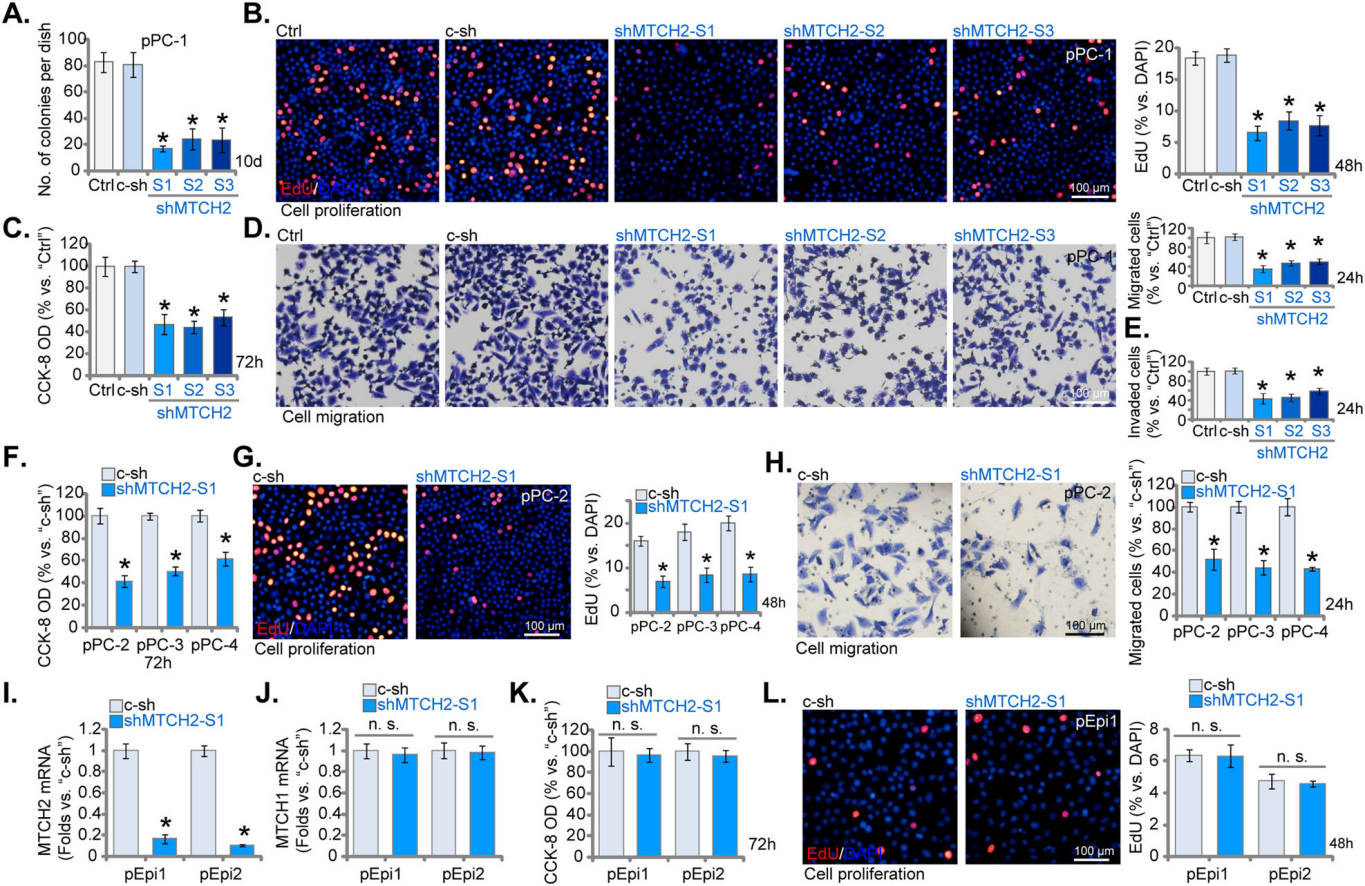
MTCH2 silencing inhibits the proliferative and migratory capacities of primary human CRPC cells. The primary pPC-1 cells were subjected to individual treatments with specific MTCH2 shRNAs (shMTCH2-S1, shMTCH2-S2, or shMTCH2-S3, each representing a unique sequence) or a control scramble non-sense shRNA (c-sh), the stable cells were formed after puromycin selection. The equal number of cells were cultured for designated time periods, and various cellular functions, including colony formation (**A**), cell proliferation (measured by the percentage of EdU-incorporated nuclei, **B**), cell viability (assessed by CCK-8 optical density, **C**), in vitro cell migration (analyzed using “Transwell” assays, **D**), and cell invasion (examined via “Matrigel Transwell” assays, **E**) were measured. Additionally, stable cells derived from other primary CRPC cells (pPC-2, pPC-3, and pPC-4) as well as the primary human prostate epithelial cells (pEpi1 and pEpi2) expressing either c-sh or shMTCH2-S1 were established and MTCH2/MTCH1 mRNA expression was examined (**I**, **J**); Equal cell numbers were cultured for designated time periods, cell viability (**F**, **K**), proliferation (**G**, **L**) and migration (**H**) were measured similarly. The data are expressed as mean ± standard deviation (SD, *n* = 5). “Ctrl” denotes the parental control cells. Statistical significance is marked by *P < 0.05 when compared to “c-sh” cells. “n.s.” denotes non-statistically significant differences (***P*** > 0.05). The experiments depicted in this figure were conducted five times (biological replicates), consistently producing similar results. The scale bar represents 100 μm.

The depletion of MTCH2 using the specified shRNAs mentioned above (see Fig. 4) significantly hindered the proliferation and colony formation abilities of pPC-1 cells (Fig. 5A) and reduced the proportion of EdU-positive nuclei (Fig. 5B). Furthermore, cell viability, assessed by the CCK-8 assay, was diminished in MTCH2-silenced pPC-1 cells (Fig. 5C). Additionally, MTCH2 knockdown substantially impaired the motility of pPC-1 cells, resulting in marked reductions in both in vitro migration (Fig. 5D) and invasion (Fig. 5E) capacities, as determined by the “Transwell” and “Matrigel Transwell” assays, respectively. It is important to note that the control shRNA (c-sh) did not significantly alter the functional properties of pPC-1 cells (Fig. 5A–E).

Consistent with these observations in pPC-1 cells, MTCH2 silencing using shMTCH2-S1 significantly reduced cell viability (CCK-8 OD, Fig. 5F) in other primary human CRPC cells, including pPC-2, pPC-3, and pPC-4. It also suppressed cell proliferation (EdU incorporation, Fig. 5G) and decreased cell migration (Fig. 5H) in these primary CRPC cells. In primary human prostate epithelial cells (pEpi1 and pEpi2, as reported in an earlier study [22]), treatment with shMTCH2-S1 led to a substantial reduction in *MTCH2* mRNA levels (Fig. 5I, J), while *MTCH1* mRNA levels remained unaffected (Fig. 5I, J). However, MTCH2 silencing did not affect the viability (CCK-8 OD, Fig. 5K) or proliferation (nuclear EdU incorporation, Fig. 5L) in non-cancerous epithelial cells. These findings underscore the crucial role of MTCH2 in sustaining the proliferative and migratory capabilities of CRPC cells.

### MTCH2 silencing provokes apoptosis activation in primary human CRPC cells

**Fig. 6:**
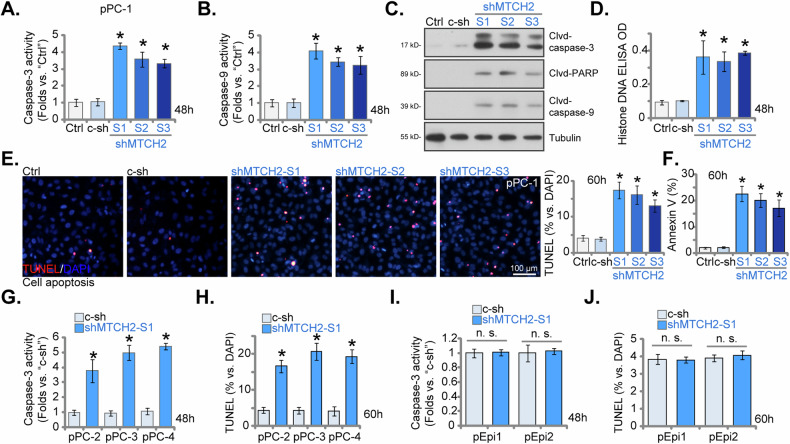
MTCH2 silencing provokes apoptosis activation in primary human CRPC cells. The primary pPC-1 cells were subjected to individual treatments with specific MTCH2 shRNAs (shMTCH2-S1, shMTCH2-S2, or shMTCH2-S3, each representing a unique sequence) or a control scramble non-sense shRNA (c-sh), stable cells were formed after puromycin selection. Equal cell numbers were cultured for designated time periods, the Caspase-3 activity (**A**), the Caspase-9 activity (**B**), expression levels of apoptosis-related proteins (**C**), and the Histone-DNA contents (**D**) were tested, with cell apoptosis measured via the nuclear TUNEL staining (**E**) and the Annexin V FACS (**F**) assays. Additionally, stable cells derived from other primary CRPC cells (pPC-2, pPC-3, and pPC-4), as well as the primary human prostate epithelial cells (pEpi1 and pEpi2) expressing either c-sh or shMTCH2-S1, were established and the equal number of cells were cultured for the designated time periods, the Caspase-3 activity (**G**, **I**) and cell apoptosis (elevated via the nuclear TUNEL staining, **H**, **J**) were tested similarly. The data are expressed as mean ± standard deviation (SD, *n* = 5). “Ctrl” denotes the parental control cells. Statistical significance is marked by *P < 0.05 when compared to “c-sh” cells. “n.s.” denotes non-statistically significant differences (***P*** > 0.05). The experiments depicted in this figure were conducted five times (biological replicates), consistently producing similar results. The scale bar represents 100 μm.

The shRNA-mediated knockdown of MTCH2 exhibited pronounced inhibitory effects on mitochondrial function, cell viability, proliferation, and migration in primary CRPC cells. To further elucidate the functional implications of MTCH2, its impact on cell apoptosis was assessed. The primary pPC-1 cells treated with shMTCH2-S1, shMTCH2-S2, or shMTCH2-S3 showed a significant increase in Caspase-3 activity (Fig. 6A) and Caspase-9 activity (Fig. 6B). Additionally, MTCH2 silencing via shRNA led to the cleavage of Caspase-3, Caspase-9, and Poly(ADP-ribose) polymerase-1 (PARP-1) in these cells (Fig. 6C). Furthermore, levels of histone-bound DNA, an important marker of apoptosis activation, were elevated in MTCH2 shRNA-expressing pPC-1 cells (Fig. 6D). This cascade of events, triggered by MTCH2 knockdown, culminated in heightened apoptosis in pPC-1 cells, evidenced by a significant increase in TUNEL-positive nuclei (Fig. 6E) and a higher percentage of Annexin V-positive pPC-1 cells (Fig. 6F). In contrast, the control shRNA (c-sh) treatment did not provoke apoptosis in pPC-1 cells (Fig. 6A–F).

Moreover, in additional primary human CRPC cells (pPC-2, pPC-3, pPC-4), the establishment of stable MTCH2 knockdown using the shMTCH2-S1-expressing lentiviral vector (as depicted in Figs. 4, 5) similarly induced Caspase-3 activation (Fig. 6G) and increased the number of TUNEL-positive nuclei (Fig. 6H), thus confirming the induction of apoptosis across multiple cancer cells. In epithelial cells, pEpi1 and pEpi2, MTCH2 knockdown (see Fig. 5) using shMTCH2-S1 did not significantly increase Caspase-3 activity (Fig. 6I) or nuclear TUNEL staining (Fig. 6J), again supporting a cancer cell-specific effect by MTCH2 knockdown. These findings underscore the critical role of MTCH2 in regulating apoptotic pathways in CRPC cells.

### MTCH2 knockout impairs mitochondrial function and inhibits CRPC cell progression

**Fig. 7:**
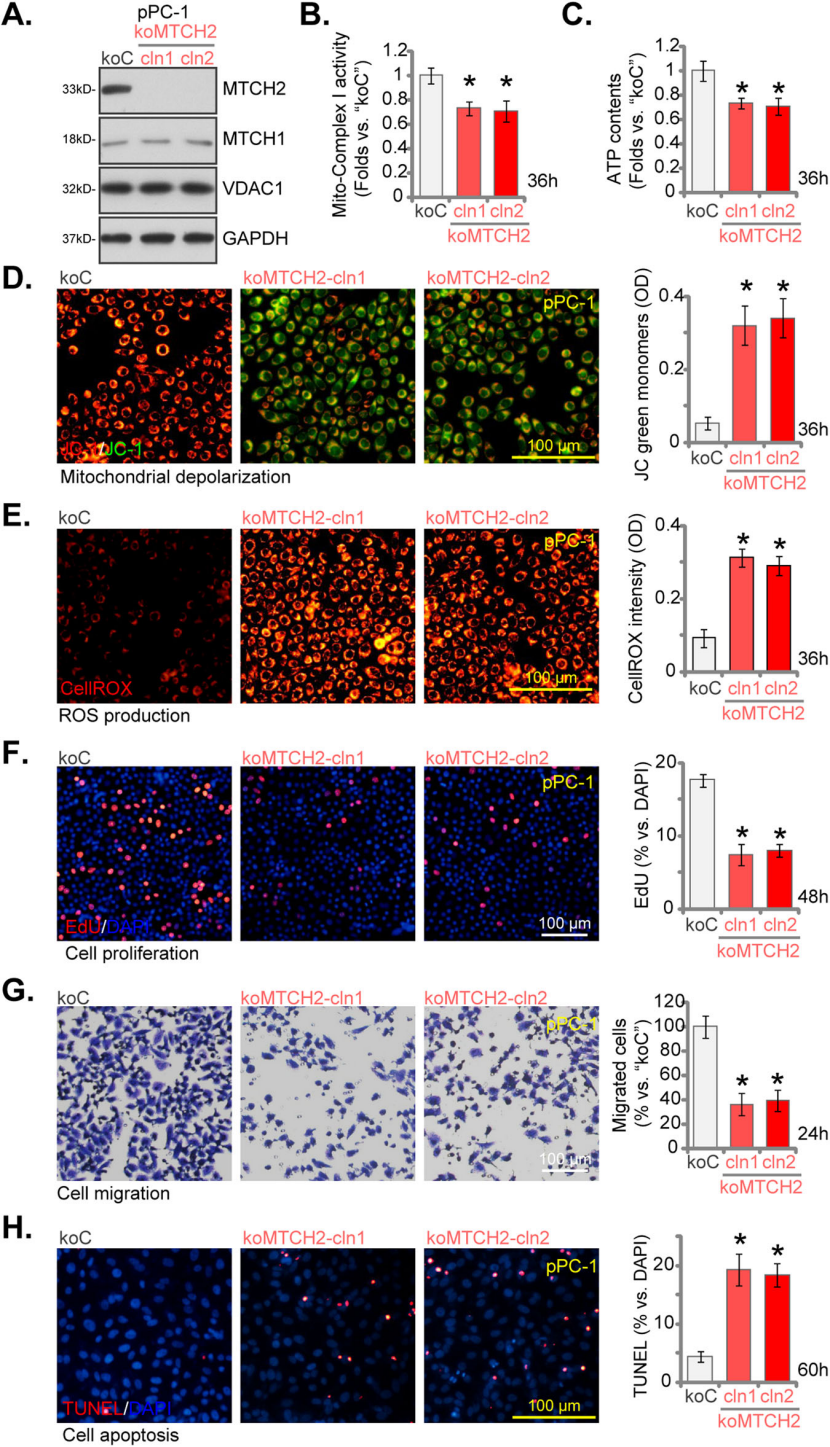
MTCH2 knockout impairs mitochondrial function and inhibits CRPC cell progression. The single stable pPC-1 cells with a Cas9 construct and the CRISPR/Cas9-MTCH2 KO construct (koMTCH2-cln1, or koMTCH2-cln2, representing two single-cell colonies) or the CRISPR/Cas9-KO vector (“koC”) were cultured and the expression levels of MTCH1 and MTCH2 were assessed using Western blotting (**A**). The equal number of cells were cultured for designated time periods, the mitochondrial complex I activity (**B**), ATP contents (**C**) were tested. Mitochondrial depolarization was determined by JC-1 green monomer intensity (**D**), and the ROS levels were measured via quantifying CellROX fluorescence intensity (**E**); Cell proliferation (measured by the percentage of EdU-incorporated nuclei, **F**), in vitro cell migration (analyzed using “Transwell” assays, **G**) and apoptosis (measured via nuclear TUNEL staining, **H**) were tested. The data are expressed as mean ± standard deviation (SD, *n* = 5). Statistical significance is marked by *P < 0.05 when compared to “koC” cells. The experiments depicted in this figure were conducted five times (biological replicates), consistently producing similar results. The scale bar represents 100 μm.

To exclude the potential off-target effects associated with the shRNAs and to ensure a complete knockout (KO) of MTCH2, we utilized CRISPR/Cas9-based methodology. Specifically, a lentivirus expressing the CRISPR/Cas9-MTCH2 KO construct along with a puromycin resistance gene was transduced into Cas9-expressing stable pPC-1 cells. Following puromycin selection, individual stable clones were isolated and subjected to comprehensive MTCH2 KO screening, resulting in the generation of single-cell-derived pPC-1 MTCH2 KO colonies: koMTCH2-cln1 and koMTCH2-cln2. Western blotting analysis verified the total ablation of MTCH2 protein expression in these koMTCH2 pPC-1 cells (Fig. 7A). MTCH1 and VDAC1 protein levels remained unaltered (Fig. 7A), indicating the specificity of the KO procedure. Consistent with the findings observed in MTCH2-silenced cells, there was a marked decrease in the activity of mitochondrial complex I (Fig. 7B) as well as ATP contents (Fig. 7C) within MTCH2 KO pPC-1 cells. Additionally, the KO of MTCH2 resulted in mitochondrial depolarization, evidenced by the increase of JC-1 monomers (Fig. 7D). The heightened intensity of CellROX staining implied an increase in ROS production within MTCH2 KO CRPC cells (Fig. 7E).

Subsequent experiments provided robust evidence that MTCH2 KO significantly impeded the proliferation of pPC-1 cells, as indicated by a marked reduction in EdU-positive nuclei staining (Fig. 7F). Moreover, the assays assessing in vitro cell migration demonstrated that koMTCH2-cln1 and koMTCH2-cln2 pPC-1 cells exhibited markedly reduced migration capabilities (Fig. 7G). MTCH2 KO also triggered apoptosis in pPC-1 cells, as shown by an increased nuclear TUNEL ratio (Fig. 7H). Collectively, these findings again underscore the pivotal role of MTCH2 in preserving mitochondrial integrity and regulating essential cellular functions in CRPC cells.

### MTCH2 overexpression enhances the mitochondrial function and augments aggressive cellular behaviors in CRPC cells

**Fig. 8:**
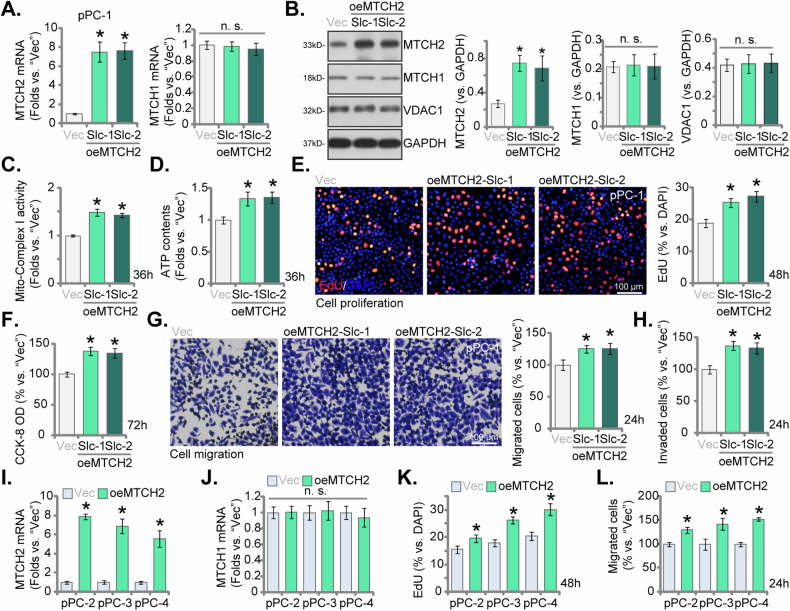
MTCH2 overexpression enhances the mitochondrial function and augments aggressive cellular behaviors in CRPC cells. The primary pPC-1 cells were transduced with a lentiviral construct expressing MTCH2, resulting in the creation of two stable cell selections, namely oeMTCH2-Slc-1 and oeMTCH2-Slc-2, following selection and verification of overexpression. As a control, pPC-1 cells were stably transduced with an empty vector (designated as “Vec”). The expression levels of MTCH1 and MTCH2 were subsequently evaluated (**A**, **B**). The equal number of cells were cultured for designated time periods, the mitochondrial complex I activity (**C**), ATP contents (**D**) were tested. Cell proliferation (measured by the percentage of EdU-incorporated nuclei, **E**), cell viability (CCK-8 OD, **F**), in vitro cell migration (analyzed using “Transwell” assays, **G**) and invasion (examined via “Matrigel Transwell” assays, **H**) were also tested. The primary human CRPC cells derived from three other patients, pPC-2/pPC-3/pPC-4, were also stably transduced with the lentiviral MTCH2-expressing construct (“oeMTCH2”) or the empty vector (“Vec”), *MTCH1/2* mRNA expression was measured (**I**, **J**); Equal cell numbers were cultured for designated time periods, cell proliferation (**K**) and in vitro migration (**L**) were measured similarly, with results quantified. The data were expressed as mean ± standard deviation (SD, *n* = 5). Statistical significance is marked by *P < 0.05 when compared to “Vec” cells. “n.s.” denotes non-statistically significant differences (***P*** > 0.05). The experiments depicted in this figure were conducted five times (biological replicates), consistently producing similar results. The scale bar represents 100 μm.

Our research has demonstrated that silencing or knocking out MTCH2 yielded significant anti-CRPC cell effects. To investigate whether the overexpression of MTCH2 would produce contrary effects, we introduced a lentiviral MTCH2-expressing construct into primary pPC-1 cells. Post-transduction, the cells underwent puromycin selection, followed by confirmation of MTCH2 expression. This process resulted in the establishment of two distinct MTCH2-overexpressing pPC-1 cell selections, designated as “oeMTCH2-Slc-1” and “oeMTCH2-Slc-2”. Compared to the vector control cells (“Vec”), both *MTCH2* mRNA (Fig. 8A) and protein (Fig. 8B) levels were significantly elevated in oeMTCH2-Slc-1 and oeMTCH2-Slc-2 pPC-1 cells. Conversely, the expression levels of *MTCH1* mRNA (Fig. 8A) and protein (Fig. 8B) remained unaltered. The expression of the mitochondrial protein VDAC1 was also unaltered (Fig. 8B).

Functional analyses demonstrated that the ectopic overexpression of MTCH2 led to an enhancement in the activity of mitochondrial complex I (Fig. 8C) and a concomitant increase in ATP levels (Fig. 8D) within pPC-1 cells. This overexpression promoted cellular proliferation, as evidenced by a higher ratio of EdU-positive nuclei (Fig. 8E). Cell viability, as measured by the CCK-8 assay, was elevated in MTCH2-overexpressing pPC-1 cells (Fig. 8F). Additionally, MTCH2 overexpression significantly accelerated both in vitro cell migration (Fig. 8G) and invasion (Fig. 8H) in pPC-1 cells. In primary pPC-2, pPC-3, and pPC-4 cells, stable transduction with the lentiviral MTCH2-expressing construct led to a significant upregulation of *MTCH2* mRNA levels (denoted as “oeMTCH2”, Fig. 8I), while *MTCH1* mRNA expression remained unaffected (Fig. 8J). Overexpression of MTCH2 enhanced cell proliferation, as indicated by increased EdU incorporation (Fig. 8K), and accelerated in vitro cell migration (Fig. 8L) in these other primary CRPC cells. These findings collectively underscore the potent role of MTCH2 in enhancing mitochondrial function and driving aggressive cellular behaviors in CRPC cells.

### MTCH2 silencing disrupts the mitochondrial function and hinders the growth of CRPC xenografts in nude mice

**Fig. 9:**
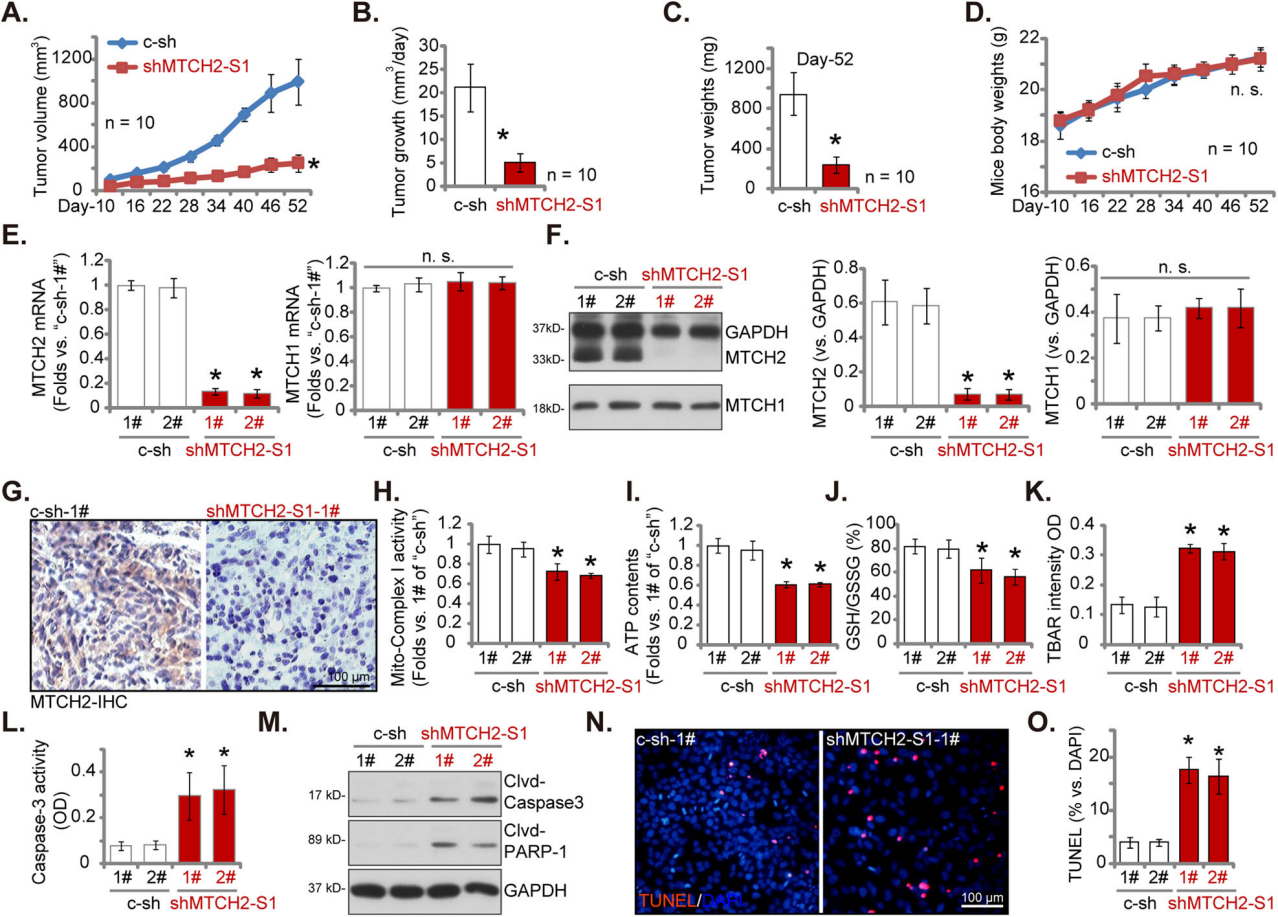
MTCH2 silencing disrupts the mitochondrial function and hinders the growth of CRPC xenografts in nude mice. Stable pPC-1 cells expressing MTCH2 shRNA (“shMTCH2-S1”) or control shRNA (“c-sh”) were subcutaneously injected into the flanks of nude mice, with each mouse receiving eight million cells. Each experimental group included ten mice (*n* = 10). Observations commenced ten days post-injection (“Day 10”). Tumor volumes (**A**, in mm^3^) and mouse body weights (**D**, in grams) were measured every 6 days from Day 10 to Day 52. The daily growth rate of the xenografts was evaluated (**B**). On Day 52, the xenografts were surgically excised and weighed (**C**). For further analysis, two xenografts from each group (labeled as “1#” and “2#”) were selected for protein and mRNA expression analyses in the tissue lysates (**E**, **F**, **M**). The mitochondrial complex I activity (**H**), the ATP concentration (**I**), the ratio of GSH/GSSG (**J**), the activity of TBAR (**K**), and the activity of Caspase-3 (**L**) in the tissue lysates were assessed. Additionally, sections of the xenografts underwent immunohistochemistry (IHC) staining for MTCH2 (**G**). TUNEL/DAPI fluorescence staining was also performed in tissue sections (**N**, **O**). Data were presented as mean ± standard deviation (SD). Statistical significance was indicated by *P < 0.05 compared to the “c-sh” group. “n.s.” denotes non-statistically significant differences (***P*** > 0.05). For **A**–**D**, the sample size was ten mice per group. In **E**–**O**, each xenograft was sectioned into five pieces and analyzed individually.

To highlight the importance of MTCH2 in the in vivo growth of CRPC cells, we administered injections of eight million pPC-1 cells transduced with either shMTCH2-S1 or c-sh into the flanks of nude mice. Monitoring began 10 days post-injection (labeled as “Day 10”). The resulting tumor growth curve, with volume assessments every six days from “Day 10” to “Day 52,” revealed a marked decrease in the growth of shMTCH2-S1-expressing pPC-1 xenografts relative to c-sh controls (Fig. 9A). Analysis of the daily growth further corroborated a pronounced inhibition of tumor growth in the shMTCH2-S1 xenografts (Fig. 9B). By Day 52, all the tumors were excised and weighed, with the masses of shMTCH2-S1 pPC-1 xenografts significantly lower than those of the c-sh pPC-1 xenografts (Fig. 9C). The body weights of the mice remained consistent between both experimental groups (Fig. 9D).

For further analysis, two xenografts from each group (labeled as “1#” and “2#”) were isolated on Day 52. A marked reduction in *MTCH2* mRNA and protein expression was detected in shMTCH2-S1 pPC-1 xenograft tissue (Fig. 9E, F), while MTCH1 expression levels remained unaltered (Fig. 9E, F). The immunohistochemistry (IHC) images supported the suppression of MTCH2 protein in shMTCH2-S1 pPC-1 xenografts (Fig. 9G). Significant changes in mitochondrial function were detected in the shMTCH2-S1 pPC-1 xenograft tissue. Specifically, there were significant declines in mitochondrial complex-1 activity (Fig. 9H), ATP concentrations (Fig. 9I), and the GSH/GSSG ratio (Fig. 9J). This mitochondrial impairment was further evidenced by an elevated TBAR activity, reflecting increased lipid peroxidation, in the MTCH2-silenced xenograft tissue (Fig. 9K). There was a substantial increase in Caspase-3 activity (Fig. 9L), along with elevated levels of cleaved-Caspase-3 and cleaved-PARP-1 (Fig. 9M), in the shMTCH2-S1-expressing pPC-1 xenografts. Supporting the induction of apoptosis, tissue fluorescence staining assays demonstrated a higher percentage of TUNEL-positive nuclei in the shMTCH2-S1 pPC-1 xenograft tissue sections (Fig. 9N, O). Together, these results underscore that MTCH2 silencing disrupted mitochondrial function and hindered the growth of pPC-1 xenografts in nude mice.

## Discussion

Identifying novel targets in CRPC is crucial due to the limited efficacy of the current treatments and the aggressive nature of the disease, which often leads to poor patient outcomes. Additionally, new therapeutic targets can provide insights into the underlying mechanisms driving CRPC progression [3, 8, 29–31]. Maintaining mitochondrial function is vital for the progression of CRPC [11–15]. It has been shown that δ-tocotrienol exerted significant antitumor effects in CRPC cells by causing mitochondrial dysfunction and impairment [32]. Zhao et al. showed that PPFIA4 (type F polypeptide interacting protein alpha 4) drives CRPC progression by boosting mitochondrial metabolism via MTHFD2 (methylenetetrahydrofolate dehydrogenase 2) [33]. Cannabidiol induced cell death in hormone-refractory prostate cancer cells by altering mitochondrial bioenergetics through VDAC1 [14].

The findings of this study identify the mitochondrial protein MTCH2 as a promising therapeutic target for CRPC. The bioinformatic analyses demonstrated that MTCH2 is overexpressed in prostate cancer tissue and correlates with the key clinical parameters in patients. Single-cell sequencing further reveals elevated MTCH2 expression within prostate cancer epithelial cells. Moreover, MTCH2 is upregulated in the locally treated CRPC tissue and various primary human CRPC cells. The silencing or KO of MTCH2 significantly impaired cell viability, proliferation, and migration, while increasing apoptosis in the primary CRPC cells. Conversely, ectopic expression of MTCH2 conferred a pro-tumorigenic property to the CRPC cells, enhancing their proliferation and migration. In vivo, MTCH2 silencing significantly suppressed the growth of subcutaneous xenografts derived from primary CRPC cells in the nude mice. Thus, targeting MTCH2 presents a novel strategy to inhibit CRPC cell growth.

The prostate exhibits a distinct metabolic profile, characterized by its production and secretion of citrate for incorporation into seminal fluid. Contrary to the Warburg effect observed in many other cancers, prostate cancer demonstrates an increased oxidative phosphorylation (OXPHOS) compared to normal prostate epithelial cells [34]. This enhanced OXPHOS activity persists even in advanced and drug-resistant stages of the disease. Consequently, mitochondrial function and OXPHOS are crucial in prostate cancer progression, with their inhibition potentially leading to cellular apoptosis [34]. Therefore, the mitochondrial pathway in prostate cancer represents a particularly vulnerable target for therapeutic interventions [34].

The results of this study support that MTCH2 could be vital for mitochondrial respiration and OXHPOS in CRPC cells. We showed that MTCH2 shRNA or KO impaired the mitochondrial function, resulting in a reduced OCR, a diminished complex I activity and ATP levels, and also causing mitochondrial depolarization and increased ROS production in primary CRPC cells. Conversely, the mitochondrial complex I activity and ATP levels were augmented in the MTCH2-overexpressed CRPC cells. In vivo, elevated lipid peroxidation and decreased ATP levels were detected in the MTCH2-silenced CRPC xenografts. These studies support that MTCH2-mediated CRPC cell progression could be due to its role in supporting mitochondrial respiration and OXHPOS.

## Supplementary information

Figure S1

## Data Availability

All data were available upon request.
