# Supplementary material for: Identification of mitochondrial carrier homolog 2 as an important therapeutic target of castration-resistant prostate cancer
Source: Cell Death Dis. 2025 Feb 5;16(1):70. doi: 10.1038/s41419-025-07406-5 (PMC11799199; doi:10.1038/s41419-025-07406-5)
Supplement: Supplementary file 1 — Figure S1 [file 41419_2025_7406_MOESM1_ESM.pdf]

# Figure S1.

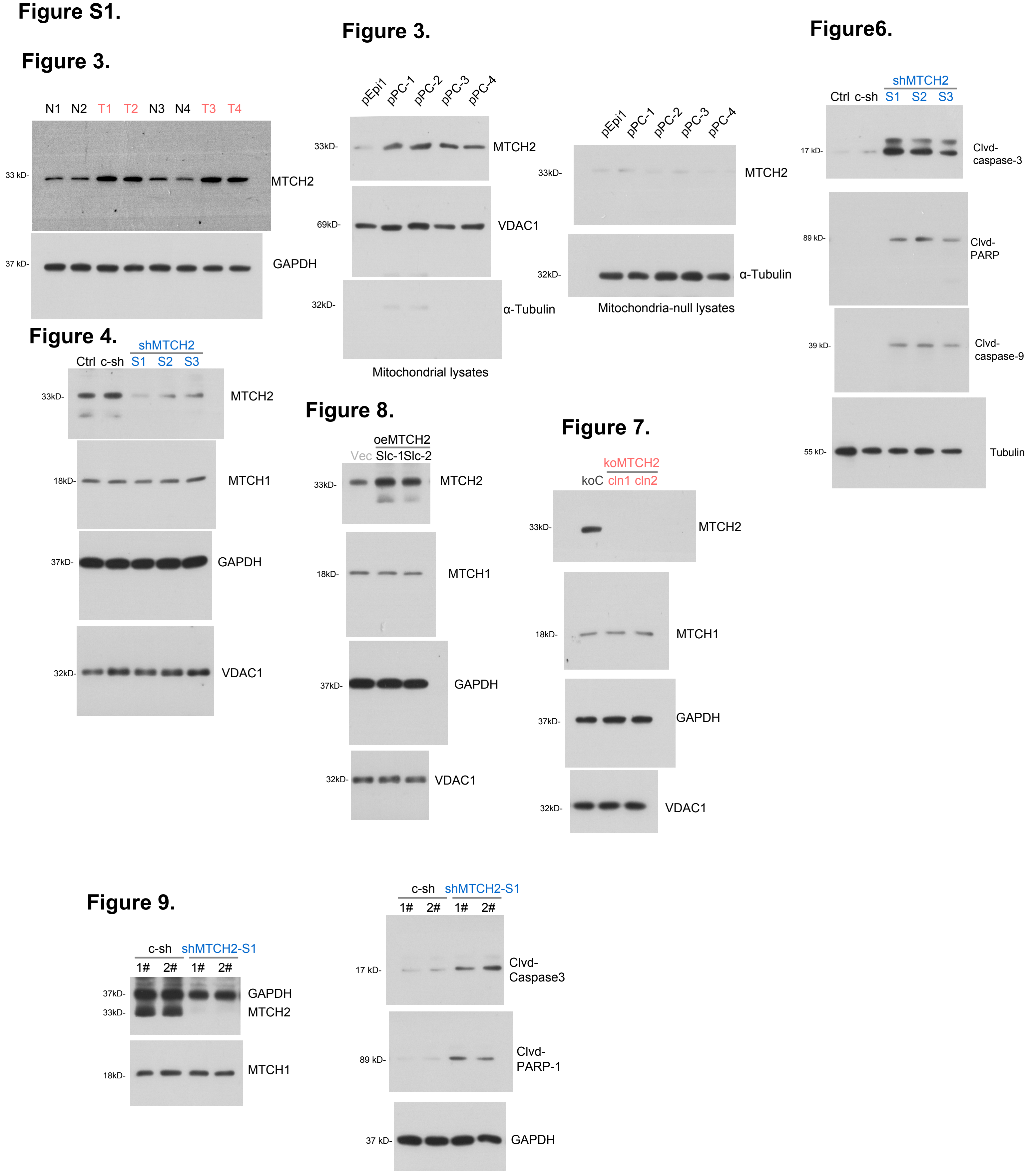


**Figure S1. The uncropped blotting images of the study.**
